# Ectopic overexpression and CRISPRi-based knockdown of *Chlamydia trachomatis* ObgE inhibits RB replication and EB reformation

**DOI:** 10.1128/jb.00282-25

**Published:** 2025-11-12

**Authors:** Colleen C. Monahan, Kiara Held, Hong Yang, Geselle Sotelo, Nicole Grieshaber, Scott Grieshaber, Anders Omsland

**Affiliations:** 1 Paul G. Allen School for Global Health, Washington State University 6760, Pullman, Washington, USA; 2 Department of Biological Sciences, University of Idaho 123394, Moscow, Idaho, USA; University of Notre Dame, Notre Dame, Indiana, USA

**Keywords:** *Chlamydia*, CRISPRi, morphological transition, replication, riboswitch

## Abstract

*Chlamydia trachomatis* is an obligate intracellular bacterium of major clinical significance. While untreated sexually transmitted infections can result in pelvic inflammatory disease and infertility, ocular infections can cause the blinding disease trachoma. During infection of host cells, *C. trachomatis* transitions between the non-replicative, infectious elementary body (EB) and the replicative, non-infectious reticulate body (RB). ObgE is a GTPase that can promote morphological differentiation in some bacteria. In *C. trachomatis*, *obgE* is maximally expressed from 16 to 24hpi, a timeframe that is associated with logarithmic growth and the onset of production of infectious progeny; therefore, ObgE is predicted to have significance during *Chlamydia* replication and/or morphological transitions. To determine the role of ObgE during the *C. trachomatis* developmental cycle, we assessed the effects of ObgE ectopic overexpression and CRISPRi-based knockdown of *obgE* on RB replication and EB formation. When ectopic overexpression of ObgE was induced, we observed a significant decrease in infectious progeny but no changes in bacterial ultrastructure. These data suggest that during ectopic overexpression of ObgE, RBs can transition into EBs; however, EBs are diminished in their ability to establish new infections. CRISPRi-based knockdown of *obgE* resulted in a 2-log decrease in bacterial yield and infectious progeny. Ultrastructural analysis revealed that knockdown of *obgE* resulted in small, underdeveloped inclusions with few cells inside. In total, while ectopic overexpression of ObgE negatively affects production of infectious EBs, CRISPRi-based knockdown of *obgE* severely affects RB replication, inclusion development, and generation of EBs.

**IMPORTANCE:**

The pathogenesis of *C. trachomatis* is reliant on the transition between the non-replicative, infectious elementary body (EB) and the replicative, non-infectious reticulate body (RB). Therefore, understanding the molecular determinants of *Chlamydia* developmental transitions is of the utmost importance. ObgE has been shown to regulate morphological transitions in other bacteria and is thus predicted to have relevance during regulation of the *Chlamydia* developmental cycle. Using both ectopic overexpression and CRISPRi-based knockdown of ObgE/*obgE*, we identify the significance of balanced ObgE expression for RB replication and the formation of infectious EBs. Our findings further expand our knowledge of how developmental transitions in *Chlamydia* are regulated.

## INTRODUCTION

*Chlamydia trachomatis* is an obligate intracellular bacterial pathogen with distinct pathovariants (i.e., serovars) within the species associated with blinding ocular or sexually transmitted infections (1). Upon invasion of a host cell, *C. trachomatis* transitions between the non-replicative, infectious elementary body (EB) and the replicative, non-infectious reticulate body (RB) inside of a vacuole termed the chlamydial inclusion (2). The RB then asynchronously transitions back to the EB, allowing a new round of infection to occur. Recent evidence suggests that generation of the EB occurs via a transitional cell form, the intermediate body (IB) (3, 4). These distinct cell forms possess unique metabolic features (5, 6) and gene expression patterns (2, 7). Developmental transitions in *Chlamydia* rely on temporally controlled gene expression, as evidenced by abnormal development of *C. trachomatis* strains with defects in specific genes (8).

In this study, we assess the significance of ObgE (*CTL0675*, aka, *cgtA/yhbZ*), a highly conserved GTPase, in chlamydial replication and/or developmental transitions. In other bacteria, ObgE has been linked to chromosome segregation (9, 10), control of replication (11), ribosomal maturation (12, 13), lipopolysaccharide synthesis (14), regulation of the stringent response (12), and morphological differentiation (15–17). While the function of ObgE in *Escherichia coli* and some other bacteria has been characterized, the function of ObgE during chlamydial replication and developmental transitions remains unclear.

In *C. trachomatis*, *obgE* is maximally expressed from 16 to 24hpi (18), a timeframe that correlates with log-phase growth of the organism and the initiation of EB reformation (2). We utilized ectopic overexpression and CRISPRi-based knockdown of ObgE/*obgE* in *C. trachomatis* serovar L2/434/Bu to test the effects of manipulating ObgE expression on replication and production of infectious EBs. We identify a regulatory role for ObgE during *C. trachomatis* replication and EB reformation, consistent with the need for tight regulation of ObgE expression for optimal virulence. Additionally, we describe a role for ObgE in the regulation of virulence factor secretion, as specific genes related to the chlamydial type III secretion system (T3SS) were abnormally expressed upon manipulation of ObgE expression.

## RESULTS

### Ectopic overexpression of ObgE reduces generation of infectious EB progeny

In *E. coli* and *Caulobacter crescentus*, *obgE* has been identified as an essential gene (19, 20), suggesting that *obgE* may also be essential in *C. trachomatis*. Accordingly, we probed the effects of ectopic overexpression of ObgE on replication and the generation of infectious EB progeny by transforming *C. trachomatis* serovar L2/434/Bu with pBOMB4::R-ObgE-3xFLAG to generate the strain R-ObgE (Fig. S1A). Expression of ObgE-3xFLAG is under the control of the theophylline-inducible E riboswitch. As a negative control, *C. trachomati*s was also transformed with pBOMB4::R-Clover-3xFLAG to generate the strain R-Clover (Fig. S1A). Expression of ObgE-3xFLAG and Clover-3xFLAG was verified by Western blot (24hpi) (Fig. S1B), and expression of ObgE-3xFLAG was additionally validated by immunofluorescence analysis during infection of HeLa cells (Fig. S1C). The expression of ObgE-3xFLAG and Clover-3xFLAG is dependent on the concentration of theophylline.

**Fig 1:**
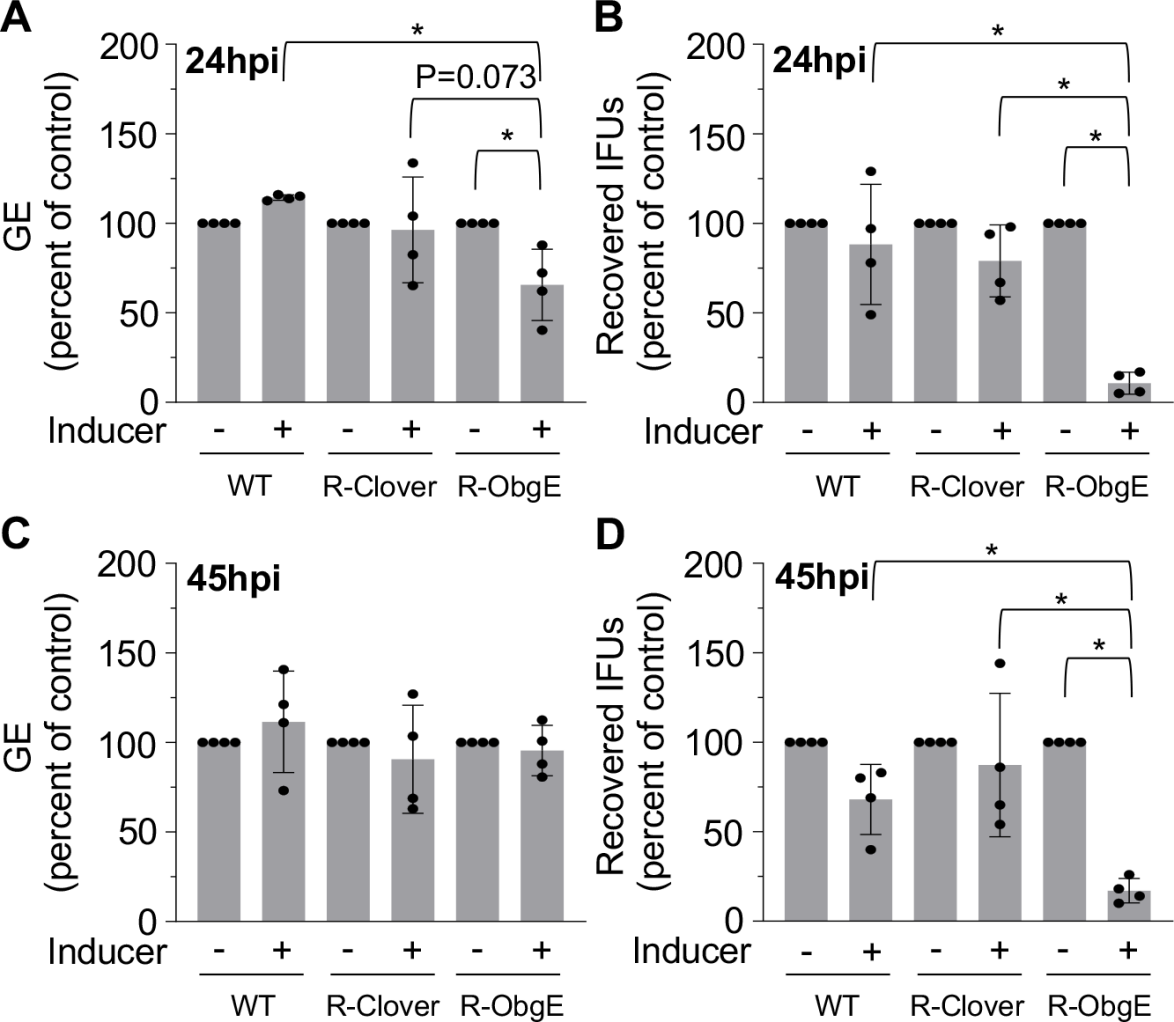
Ectopic overexpression of ObgE results in reduction of recovered IFUs. (**A**) Ectopic expression of ObgE reduced GE by 35% at 24hpi when compared to the non-induced R-ObgE control. (**B**) IFU assays showed a 90% decrease in recovered IFUs at 24hpi when compared to the non-induced R-ObgE control. (**C**) Ectopic expression of ObgE had no effect on GE at 45hpi when compared to the non-induced R-ObgE control. (**D**) IFU assays showed an 83% decrease in recovered IFUs at 45hpi when compared to the non-induced R-ObgE control. Bars = mean ± SD (*N* = 4). Statistical significance was calculated using one-way ANOVA with Tukey’s post-hoc test. An asterisk indicates statistical significance with *P* < 0.05. The inoculum for IFU assays was normalized to GE.

To test if ectopic overexpression of ObgE affects *C. trachomatis* replication and/or developmental transitions, R-ObgE was used to infect HeLa cell cultures. Bacteria were harvested from infected host cells at 24 and 45hpi for analysis of genome equivalents (GE), a qPCR-based measure of the bacterial population based on genome counts, while production of infectious EBs was measured by an inclusion forming unit (IFU) assay (21). Each IFU assay was normalized to GE, allowing enumeration of recovered infectious EBs per total cell count. The addition of 0.1 mM theophylline at the time of infection had no effect on GE or recovered IFUs in HeLa cell cultures infected with wild type (WT) or R-Clover at neither 24 nor 45hpi (Fig. 1). In HeLa cells infected with R-ObgE, ectopic overexpression of ObgE-3xFLAG resulted in a moderate but statistically significant reduction in GE at 24hpi when compared to the WT control and significantly reduced the recovery of IFUs at both 24 and 45hpi when compared to the WT and R-Clover controls (Fig. 1).

To determine whether the phenotypes observed upon expression of ObgE-3xFLAG were related to a specific timeframe during the chlamydial developmental cycle, the impact of removing theophylline at various timepoints after infection was tested (Fig. S2). Theophylline was removed at timepoints correlating with completion of the EB-RB transition (6hpi), initiation of RB replication (12hpi), and ongoing EB reformation (24, 36, and 45hpi) (2) by replacing the culture medium with fresh medium lacking theophylline. When theophylline was removed at 6 or 12hpi, no effect on GE or the recovery of infectious EBs (45hpi) was observed (Fig. S2A and B). However, a significant decrease in infectious EBs occurred when theophylline was removed at 36hpi or when cultures were allowed to progress without the removal of inducer, indicating that the effects of ectopic overexpression of ObgE-3xFLAG on the recovery of IFUs are restricted to late timepoints.

### Bacterial ultrastructure is not altered by ObgE ectopic overexpression

**Fig 2:**
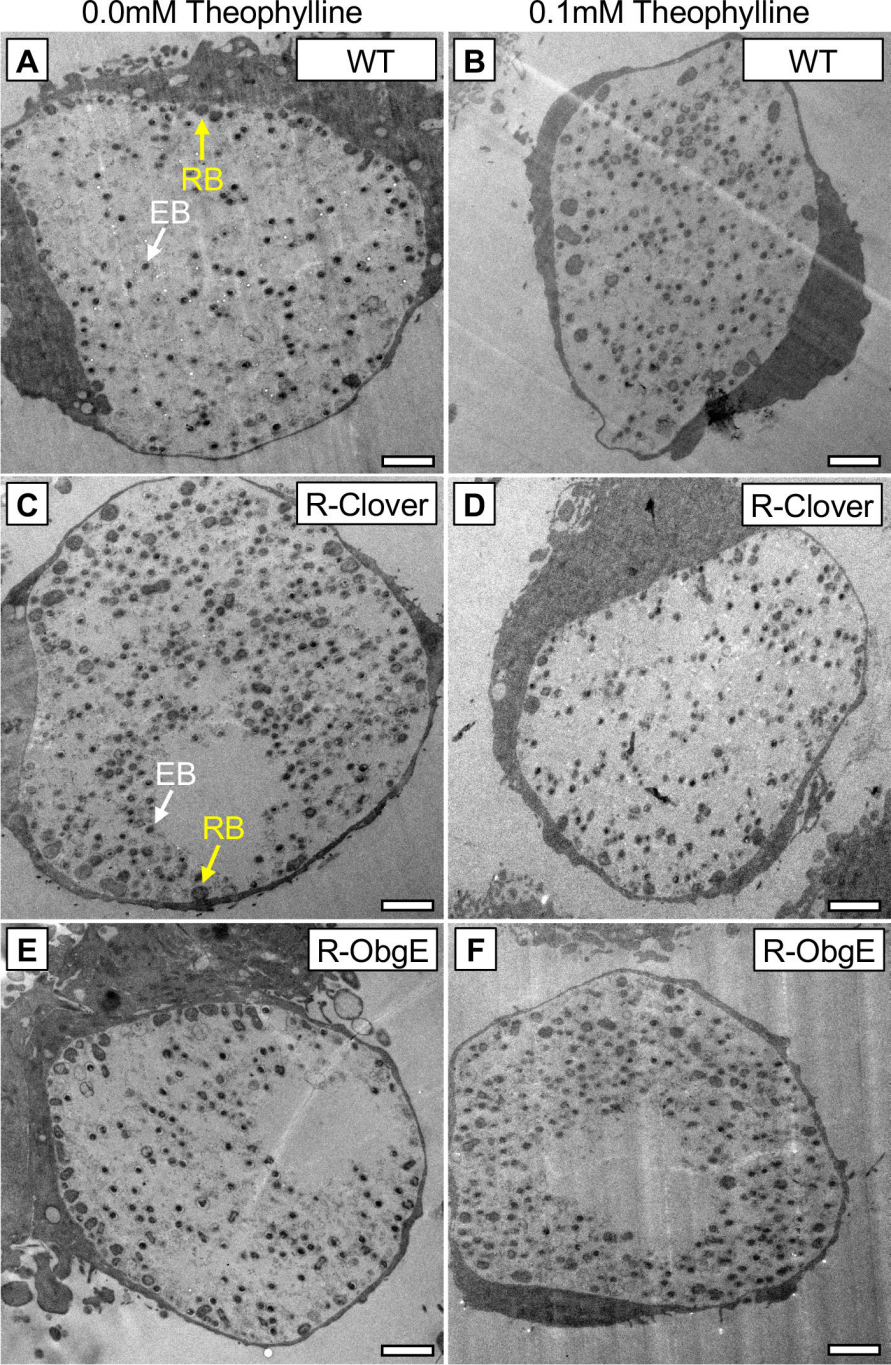
Ectopic overexpression of ObgE does not affect bacterial ultrastructure. Electron micrographs of inclusions from HeLa cells infected with (**A and B**) WT, (**C and D**) R-Clover, or (**E and F**) R-ObgE in the presence or absence of theophylline (45hpi). Theophylline was added at the time of infection. White arrows = EB. Yellow arrows = RB. Scale bars = 2 µm.

To determine whether the loss of infectious progeny observed upon ectopic overexpression of ObgE-3xFLAG was associated with alterations in morphological transitions, the ultrastructure of bacteria within inclusions was assessed by transmission electron microscopy (TEM). Induction of ObgE-3xFLAG in R-ObgE or Clover-3xFLAG in R-Clover was initiated at the time of infection, and cultures were processed for microscopy at 45hpi. Electron micrographs did not show gross changes in cell number nor morphology (Fig. 2A through F), indicating that while ectopic overexpression of ObgE-3xFLAG impairs the production of infectious progeny, the capacity of RBs to transition morphologically remains unaffected.

### Ectopic overexpression of ObgE negatively affects expression of *hctB*

**Fig 3:**
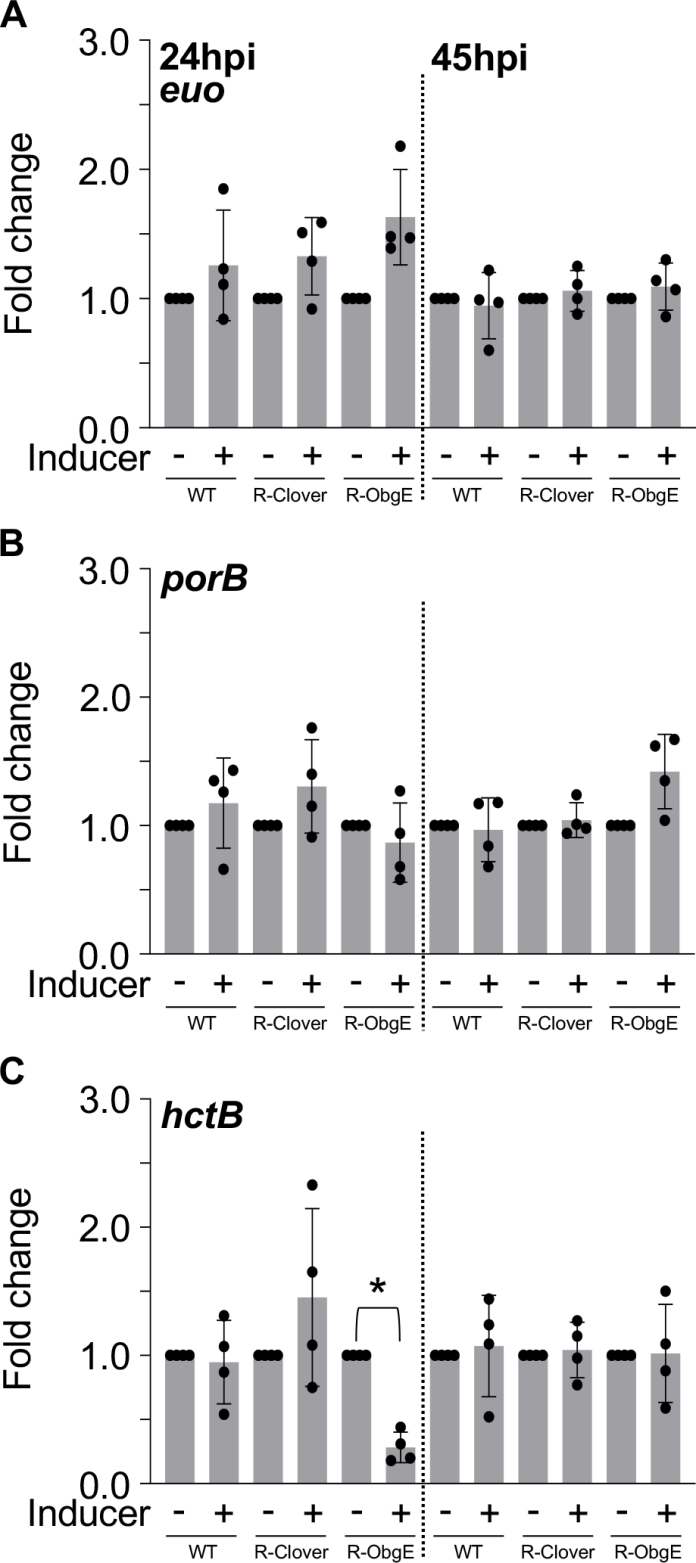
Ectopic overexpression of ObgE perturbs the expression of *hctB* at 24hpi. Transcriptional analysis of (**A**) *euo,* (**B**) *porB,* and (**C**) *hctB* during infection of HeLa cells. Bars = mean ± SD (*N* = 4). Statistical significance was calculated using one-way ANOVA with Tukey’s post-hoc test. An asterisk indicates statistical significance with *P* < 0.05 and at least a 2-fold difference in expression.

The *C. trachomatis* developmental cycle is characterized by temporally regulated gene expression. Specific genes associated with the RB (i.e., *euo*), IB (i.e., *porB*), and EB (i.e., *hctB*) can be used to track perturbations in developmental cycle kinetics in response to ectopic expression of genes (3, 22, 23). To determine if ectopic overexpression of ObgE-3xFLAG affects expression of cell form-specific genes, we performed transcriptional analysis of *euo*, *porB,* and *hctB* following infection of HeLa cells with WT, R-Clover, or R-ObgE. For each timepoint, transcripts were normalized to GE to account for differences in the number of bacteria from which RNA was extracted. Expression of *euo* and *porB* was not affected at neither 24 nor 45hpi (Fig. 3A and B). However, we observed a significant decrease in *hctB* expression (3.5-fold) in response to ectopic overexpression of ObgE-3xFLAG at 24hpi, but not at 45hpi (Fig. 3C). Despite normal levels of *hctB* in R-ObgE at 45hpi, the recovery of IFUs at 45hpi was still significantly reduced when compared to the uninduced control (Fig. 1D). In total, ectopic expression of ObgE-3xFLAG causes improper expression of *hctB*, consistent with the observed decrease in the recovery of infectious EBs.

### Expression of EB-specific T3SS genes is significantly reduced in response to ObgE ectopic overexpression

**Fig 4:**
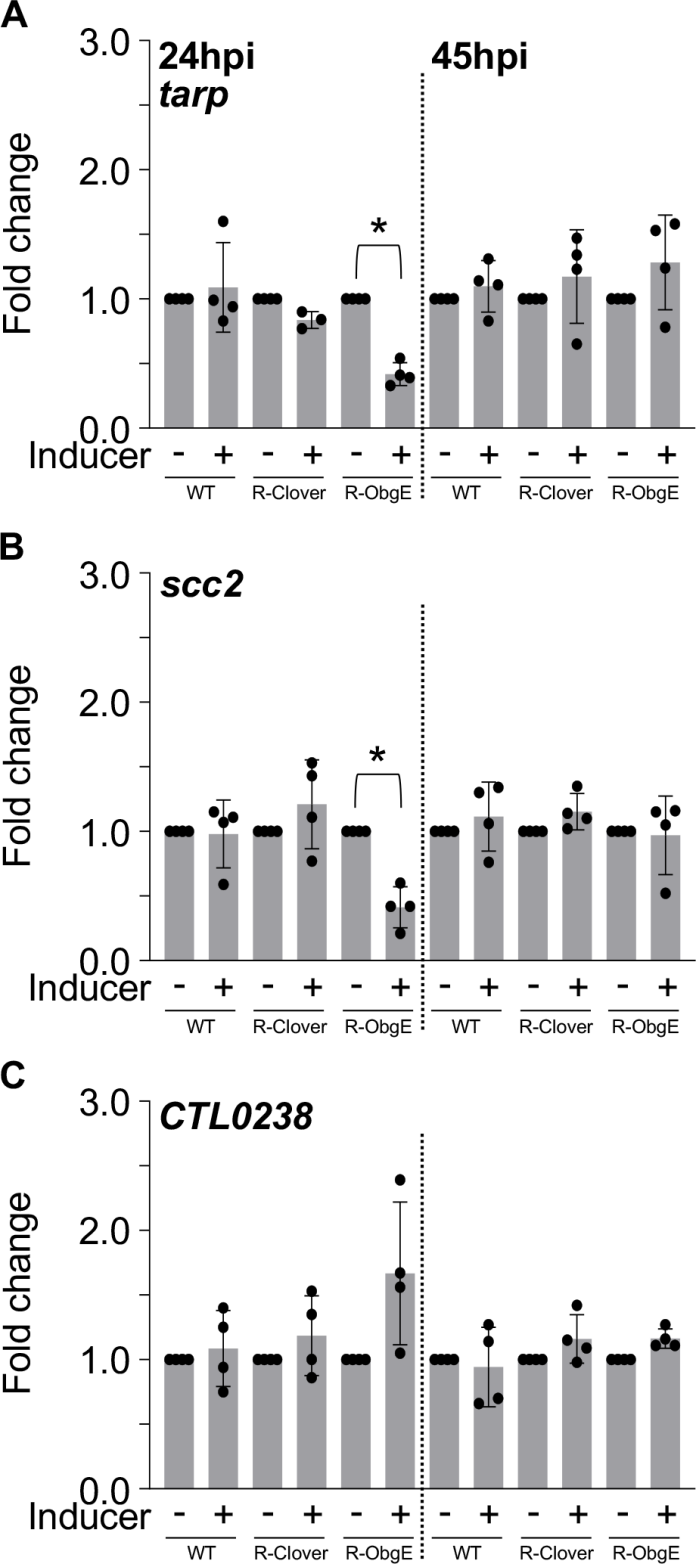
Ectopic overexpression of ObgE negatively affects transcription of EB-associated T3SS genes at 24hpi. Transcriptional analysis of (**A**) *tarp*, (**B**) *ssc2,* and (**C**) *CTL0238* during infection of HeLa cells. Bars = mean ± SD (*N* ≥ 3). Statistical significance was calculated using one-way ANOVA with Tukey’s post-hoc test. An asterisk indicates statistical significance with *P* < 0.05 and at least a 2-fold difference in expression.

The significant reduction in IFUs observed upon ectopic overexpression of ObgE-3xFLAG (Fig. 1) combined with normal bacterial ultrastructural characteristics (Fig. 2) suggest that while developmental transitions remain unaffected, EB progeny are diminished in their ability to establish new infections. This led us to investigate the expression of genes associated with the chlamydial T3SS. Effector proteins are secreted throughout the *C. trachomatis* developmental cycle and are critical for entry, establishment of the inclusion, and modulation of host–pathogen interactions (24, 25). Given the lack of obvious changes in cell form development as assessed by TEM, we analyzed whether expression of key T3SS genes was affected upon induced expression of ObgE-3xFLAG. The genes *tarp* (translocated actin recruiting phosphoprotein), *scc2* (specific *Chlamydia* chaperone 2), and *CTL0238* were selected for analysis. TARP is one of the earliest secreted proteins and recruits host-derived actin to the site of internalization (26). This effector is important for entry and inclusion stabilization. The gene *scc2* encodes a T3SS chaperone protein that assists with folding and stabilizing translocator proteins (25, 27). Expression of *tarp* and *scc2* occurs late in the developmental cycle. The gene *CTL0238* is part of a T3SS-associated operon expressed in the RB cell form (23). Transcription of *tarp* and *scc2* was significantly decreased by ~2.4-fold at 24hpi while expression of *CTL0238* was unaffected by induction of ObgE-3xFLAG expression (Fig. 4). Of note, at 45hpi, the physiological perturbations caused by overexpressing ObgE-3xFLAG remain imprinted on the recovery of infectious EBs (Fig. 1) even though expression of *tarp* and *scc2* recovered to the levels measured for the uninduced control (Fig. 4).

### Knockdown of *obgE* causes severe defects in pathogen replication and inclusion development

Ectopic overexpression of ObgE-3xFLAG resulted in loss of infectivity without having obvious effects on RB-EB transitions as assessed by analysis of pathogen ultrastructure. To investigate whether reduction in *obgE* expression affects *C. trachomatis* replication and/or formation of EBs, we used CRISPRi to knockdown expression of *obgE* via an anhydrotetracycline (aTC)-inducible (dCas12) construct. Bacteria were transformed with p2TK2-SW2::dCas12-*obgE*_gRNA-PsciEng to generate the strain dCas12-*obgE* (Fig. S3A). We confirmed that 0.5 nM aTC was sufficient to knockdown expression of *obgE* by conducting a timecourse experiment where *obgE* knockdown was induced at 5, 15, 25, and 35hpi. Expression of *obgE* was quantified 10 h after induction (15, 25, 35, and 45hpi). At all timepoints, expression of *obgE* was significantly reduced (Fig. S3B).

**Fig 5:**
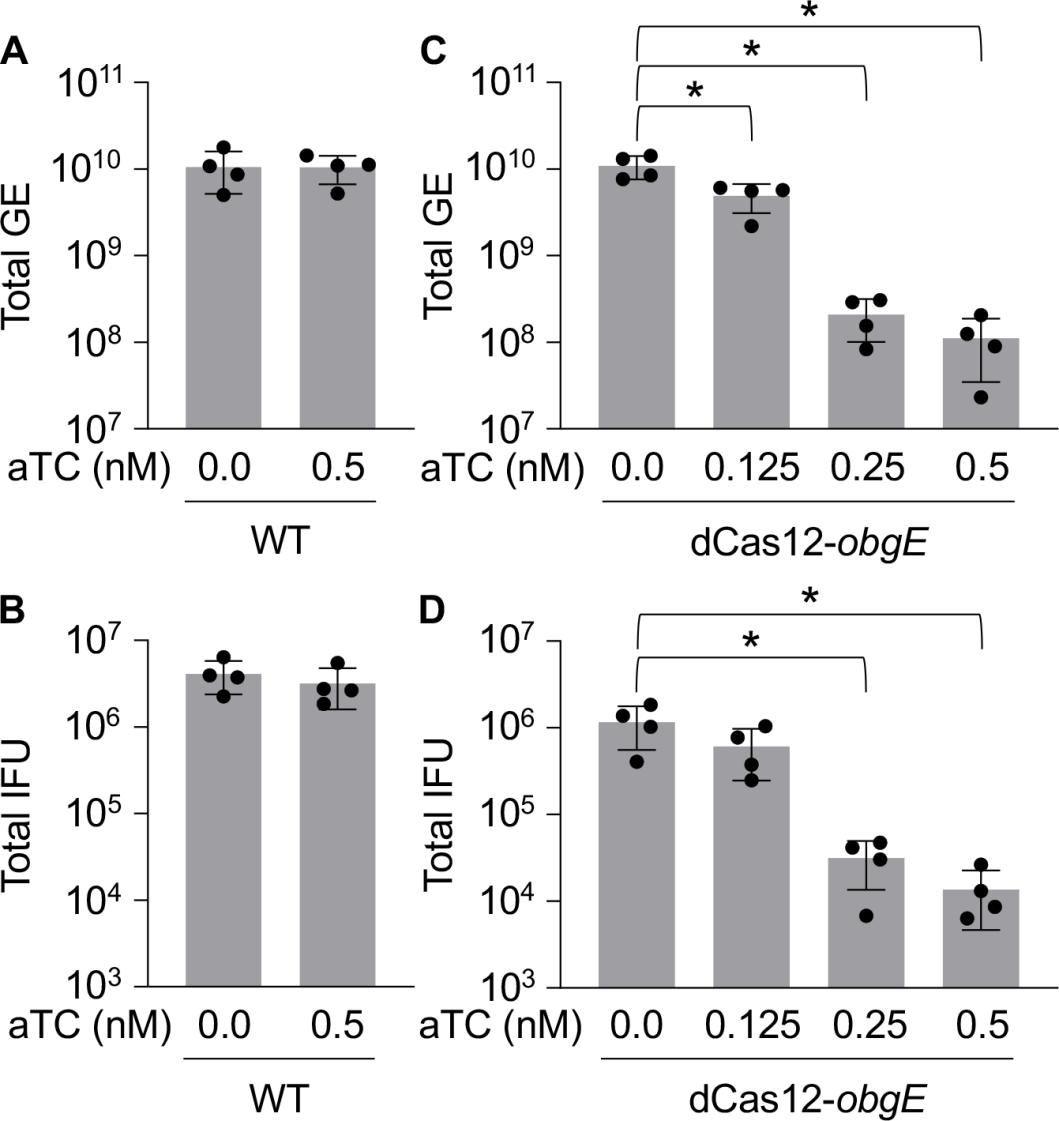
Knockdown of *obgE* displays dose-dependent effects on GE and IFU. The presence of aTC in the culture medium had no effect on (**A**) GE or (**B**) recovered IFUs in WT bacteria. Knockdown of *obgE* with increasing amounts of aTC resulted in dose-dependent decreases in (**C**) GE and (**D**) recovered IFUs at 45hpi. Bars = mean ± SD (*N* = 4). Statistical significance was calculated using Student’s *t*-test and one-way ANOVA with Tukey’s post-hoc test. An asterisk indicates statistical significance with *P* < 0.05.

**Fig 6:**
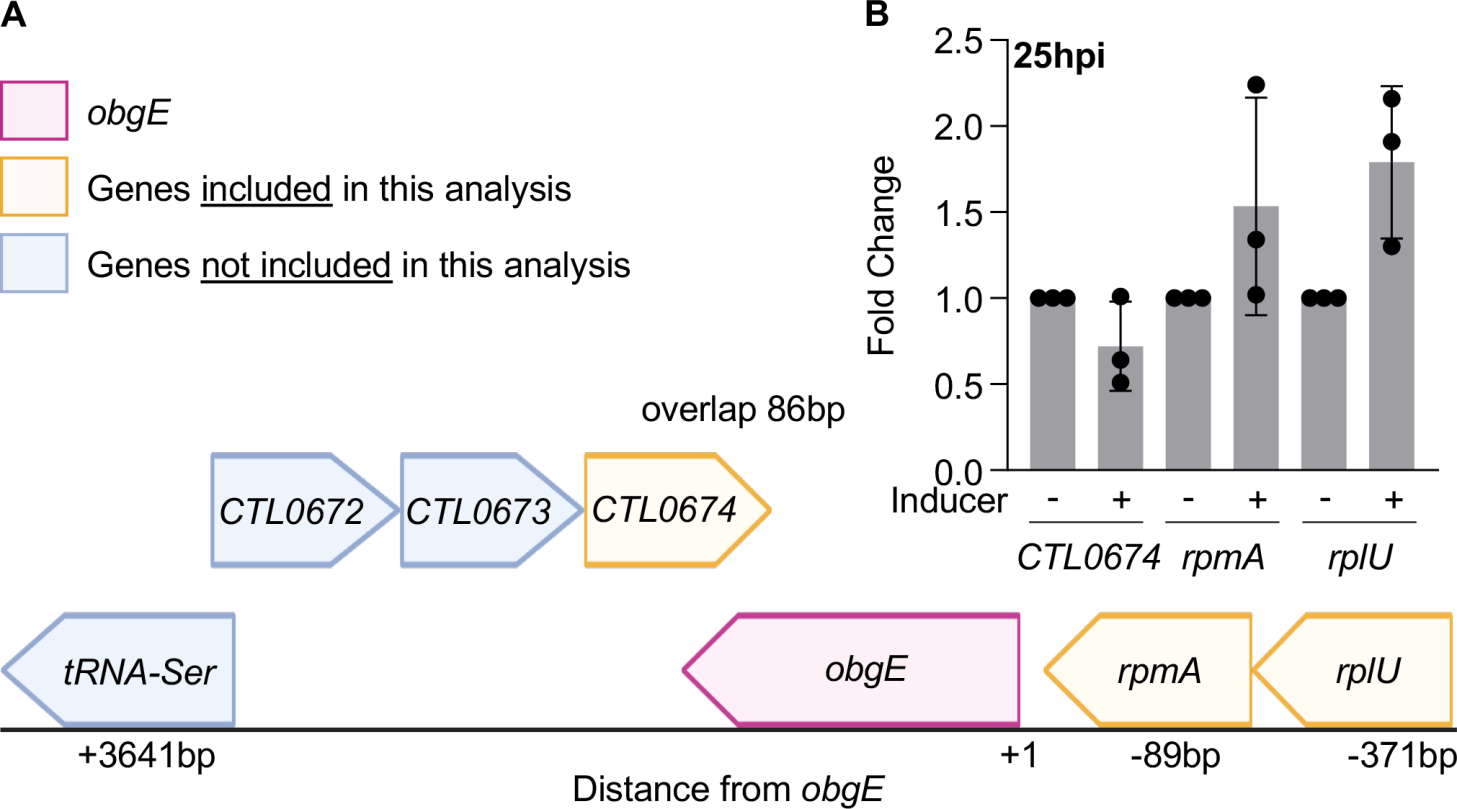
Knockdown of *obgE* does not have polar effects on nearby genes. (**A**) Schematic of the location of *obgE* in the *C. trachomatis* chromosome and the distance to nearby genes compared to the transcriptional start site of *obgE*. (**B**) Knockdown of *obgE* was induced at 15hpi and RNA was isolated at 25hpi. Knockdown of *obgE* had no significant effects on expression of *CTL0674*, *rpmA*, and *rplU*. Bars = mean ± SD (*N* = 3). Statistical significance was calculated using one-way ANOVA with post-hoc Tukey analysis (*P* < 0.05).

To determine if knockdown of *obgE* would result in dose-dependent changes in pathogen replication and/or developmental transitions, aTC was added to the culture medium in incremental amounts at the time of infection and the effects of *obgE* knockdown on GE and recovery of IFUs were assessed at 45hpi. The presence of aTC in the medium had no effects on GE or recovered IFUs for the WT control (Fig. 5A and B). However, knockdown of *obgE* resulted in dose-dependent decreases in GE and recovered IFUs (Fig. 5C and D). To ensure that the effects of *obgE* knockdown were not related to polar effects on genes adjacent to *obgE* in the genome, we quantified the effects of *obgE* knockdown on the expression of genes proximal to *obgE* (*CTL0674*, *rpmA*, *and rplU;* Fig. 6A). Knockdown of *obgE* had no effect on the expression of *CTL0674*, *rpmA*, or *rplU* (Fig. 6B). The dose-dependent phenotype and the lack of polar effects strongly suggest that the observed decreases in GE and recovered IFUs are specific to knockdown of *obgE*.

**Fig 7:**
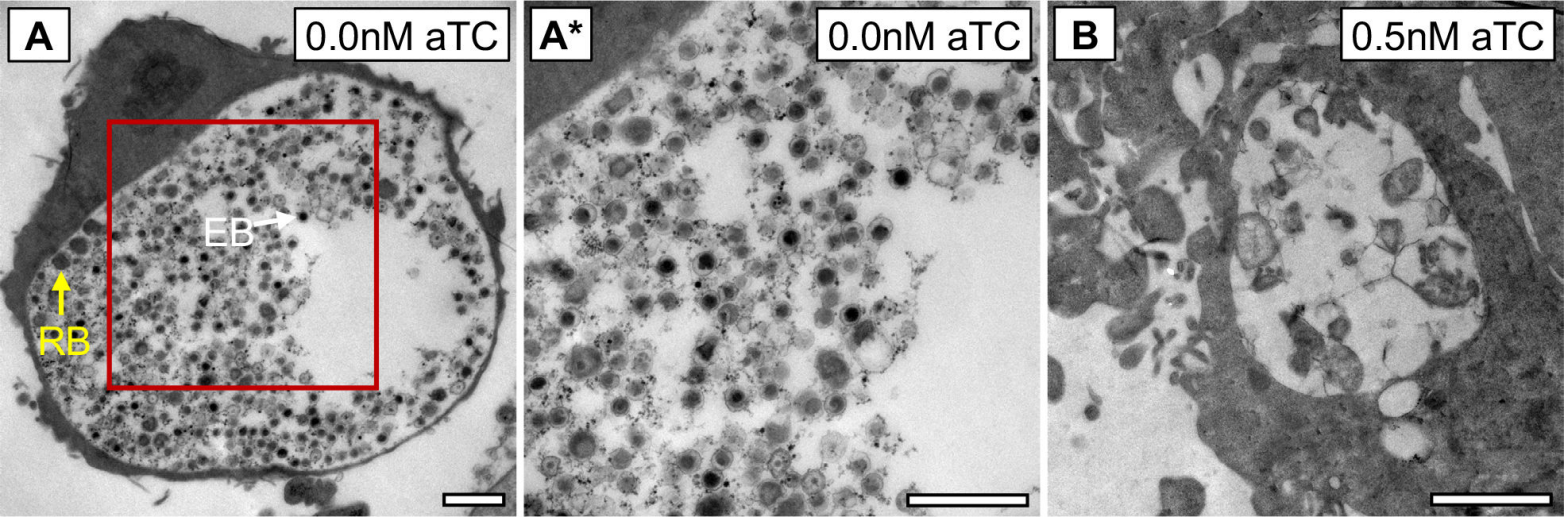
Knockdown of *obgE* affects bacterial cell morphology and inclusion development. Electron micrographs of inclusions from HeLa cells infected with dCas12-*obgE* (45hpi). aTC was added at at the time of infection. (**A**) Image of an inclusion from an infected HeLa cell cultured in the absence of aTC. (A*) Magnification of area within red border in (**A**). (**B**) Image of an inclusion from an infected HeLa cell cultured in the presence of 0.5 nM aTC. White arrows = EB. Yellow arrows = RB. Scale bars = 2 µm.

During infection of host cells, knockdown of *obgE* resulted in unusually small inclusions. Therefore, we investigated the effects of *obgE* knockdown on bacterial ultrastructure and inclusion morphology with TEM. Electron micrographs revealed that both bacterial density within the inclusion and inclusion development were severely impacted when expression of *obgE* was suppressed, as compared to the uninduced control (Fig. 7A and B). Abnormal bacterial morphology inside inclusions corresponded with a ~2-log reduction in both GE and recovered IFUs (Fig. 5C and D).

### Knockdown of *obgE* increases expression of *euo*

**Fig 8:**
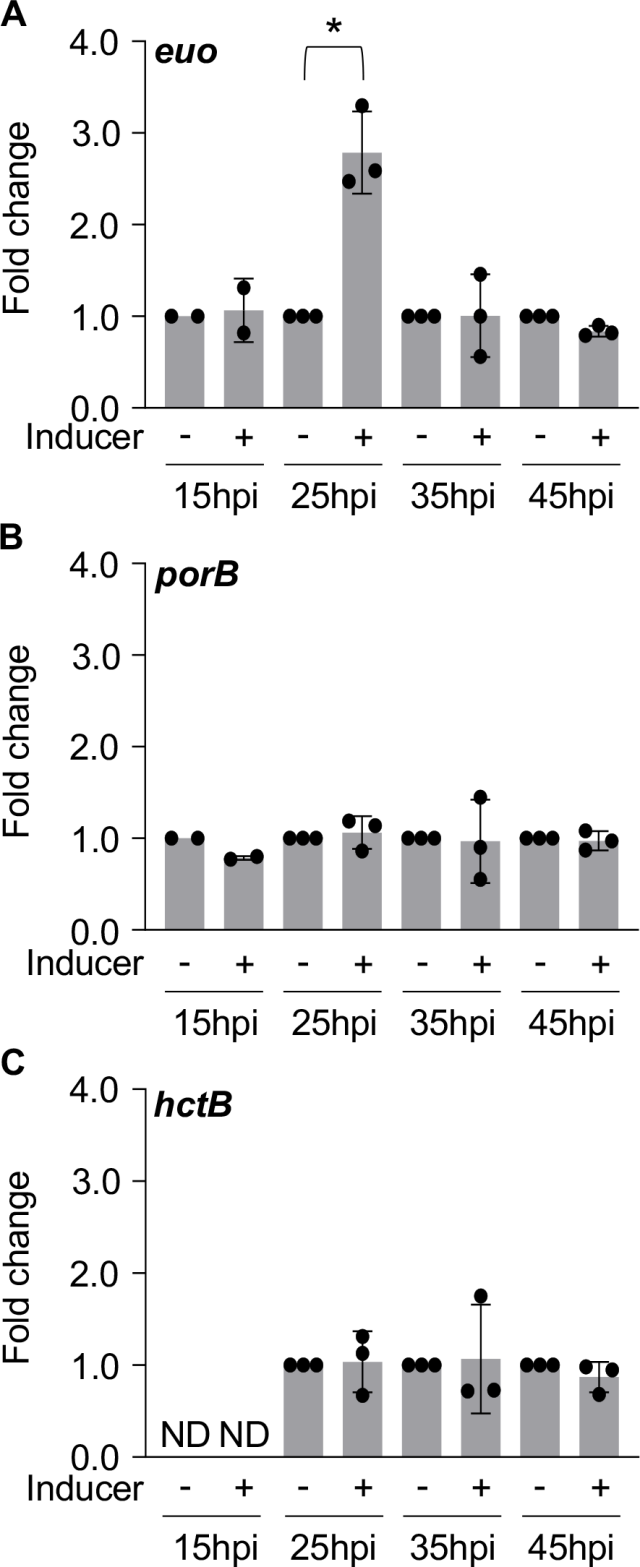
Knockdown of *obgE* results in upregulation of *euo*. Transcriptional analysis of (**A**) *euo*, (**B**) *porB*, and (**C**) *hctB* during infection of HeLa cells. Bars = mean ± SD (*N* ≥ 2). Statistical significance was calculated using one-way ANOVA with post-hoc Tukey analysis. An asterisk indicates statistical significance with *P* < 0.05 and at least a 2-fold difference in expression. ND = not detected.

In response to reduced expression of *obgE,* we observed a severe reduction in the number of bacteria inside of inclusions (Fig. 5 and 7). Accordingly, we tested if knockdown of *obgE* was associated with altered expression of *euo*, *porB*, and *hctB*. To this end, and to capture any temporal effects, knockdown of *obgE* was induced at 5, 15, 25, and 35hpi and samples were processed for RNA isolation 10 hours after induction (15, 25, 35, and 45hpi). For each timepoint, transcripts were normalized to GE to account for differences in the number of bacteria from which RNA was extracted. When knockdown of *obgE* was induced at 15hpi, expression of *euo* was upregulated 2.8-fold at 25hpi (Fig. 8A). This effect on *euo* expression was restricted to the 15–25 h timeframe, when maximal expression of *obgE* is normally observed (18). Knockdown of *obgE* did not affect expression of *porB* nor *hctB* at any timepoint tested (Fig. 8B and C). As the expression of only three genes was analyzed, we cannot discount the possibility that knockdown of *obgE* could also affect other genes typically expressed later in the developmental cycle (e.g., 35–45hpi). Since knockdown of *obgE* perturbed the expression of *euo*, a gene that is associated with the RB cell form, and significantly reduced RB replication, the effects of *obgE* knockdown appear to be primarily associated with the RB cell form.

### Knockdown of *obgE* affects expression of RB-associated T3SS effector genes

**Fig 9:**
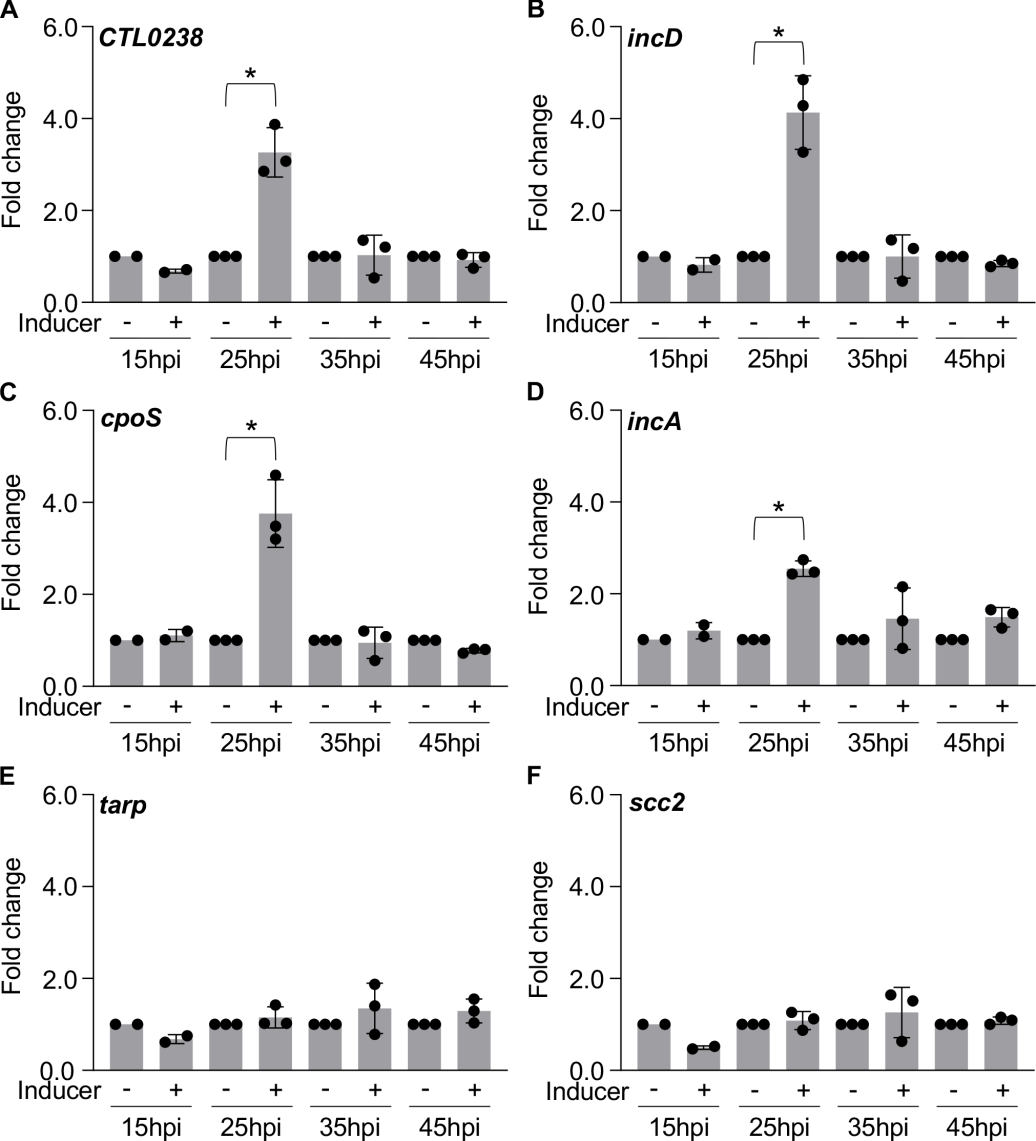
Knockdown of *obgE* results in upregulation of RB-associated T3SS genes. Transcriptional analysis of (**A**) *CTL0238*, (**B**) *incD*, (**C**) *cpoS*, (**D**) *incA,* (**E**) *tarp, and* (**F**) *scc2* following knockdown of *obgE* at 15, 25, 35, and 45hpi during infection of HeLa cells. Bars = mean ± SD (*N* ≥ 2). Statistical significance was calculated using one-way ANOVA with post-hoc Tukey analysis. An asterisk indicates statistical significance with *P* < 0.05 and at least a 2-fold difference in expression.

During infection of host cells, the *C. trachomatis* T3SS system secretes effector proteins that modulate host vesicle transport for the benefit of *C. trachomatis* (28). Vesicles that contain host-derived lipids and other macromolecules are important for inclusion maturation and bacterial replication. Electron microscopic analysis revealed that inclusion development was severely impaired in response to knockdown of *obgE*. To further elucidate why inclusion development was compromised, expression of the T3SS genes *CTL0238*, *incD*, *cpoS*, *incA*, *tarp,* and *scc2* was quantified in response to *obgE* knockdown. IncD recruits the ceramide transfer protein CERT and the ER resident protein VAPB to the inclusion membrane (29), a mechanism for *Chlamydia*-dependent acquisition of sphingomyelin (30, 31). CpoS inhibits STING-mediated pro-death signals in the host cell and promotes *C. trachomatis* intracellular survival (32). IncA mediates homotypic fusion of multiple inclusions (33) and inhibits endocytic SNARE-mediated membrane fusion (34, 35). Expression of *incA*, *incD*, *cpoS*, and *CTL0238* peaks during logarithmic replication. The effects of *obgE* knockdown on expression of RB-associated T3SS genes were restricted to 25hpi with *CTL0238, incD, cpoS*, and *incA* upregulated 3.3, 4.1, 3.8, and 2.5-fold, respectively (Fig. 9A through D). Transcription of the EB-associated T3SS genes *tarp* and *scc2* remained unchanged when compared to the uninduced control at every timepoint assessed (Fig. 9E and F).

## DISCUSSION

ObgE is an enigmatic protein that has been associated with diverse cellular processes (11, 12, 14–17, 36). Data presented herein show that overexpression of ObgE in *C. trachomatis* interferes with the establishment of fully infectious EBs via a mechanism that relates to abnormal gene expression without affecting morphological transitions. Insufficient amounts of *obgE* negatively impact the expression of RB-associated T3SS genes, which are important for RB replication and inclusion development.

Ectopic expression of ObgE moderately affected replication at 24hpi and resulted in significant decreases in the recovery of IFUs at both 24 and 45hpi. Interestingly, ectopic overexpression of ObgE-3xFLAG did not affect the ability of RBs to transition into EBs (Fig. 2) as no gross changes in bacterial morphology were observed with TEM. Ectopic expression of ObgE-3xFLAG interfered with the transcription of *hctB* and the EB-associated T3SS genes *tarp* and *scc2* (Fig. 3 and 4) at 24hpi, likely contributing to the decrease in recovered IFUs. Although expression of these late-cycle genes was unaffected at 45hpi, ectopic overexpression of ObgE-3xFLAG still resulted in a significant decrease in recovered IFUs at this late timepoint. Given that ObgE has been defined as an important anti-association factor during the assembly of ribosomal subunits in some bacteria (37), we speculate that translation of *tarp* and *ssc2* may be compromised.

Knockdown of *obgE* resulted in a ~2 log decrease in GE and recovered IFUs (Fig. 5). Additionally, the genes *euo*, *incA*, *incD*, *cpoS*, and *CTL0238* were upregulated at 25hpi in response to knockdown of *obgE* (Fig. 8 and 9). The RB-associated T3SS genes *incA, incD*, *cpoS*, and *CTL0238* are important for inclusion development and pathogen replication. For example, inhibition of CpoS results in premature cell death and cytotoxicity (32). IncD (29) and CpoS (38) mediate interaction between host proteins and the inclusion membrane, consistent with implications for pathogen replication when expression is dysregulated.

In *E. coli,* residues in the C-terminal region of ObgE were shown to be critical for mediating an interaction with the DNA-binding protein YbiB (39, 40). Primary sequence analysis of ObgE orthologs in representative obligate intracellular bacteria, facultative intracellular bacteria, and extracellular bacteria revealed that the *C. trachomatis* ObgE orthologs from serovars L2, A, and D (ObgE*_Ct_*) do not possess the C-terminal region that is essential for interaction with YbiB (Fig. S4). Notably, a BLAST search for *ybiB* in *C. trachomatis* serovar L2 revealed no related ortholog (Table S1). These two pieces of evidence strongly suggest that ObgE*_Ct_* does not function via an interaction with YbiB. While the function of ObgE is ambiguous in *Chlamydia*, the *Chlamydia abortus* ObgE ortholog (ObgE*_Ca_*) has been shown to cofractionate with the *E. coli* 50S ribosomal subunit and was confirmed to be a functional GTPase (41). ObgE*_Ct_* and ObgE*_Ca_* share high sequence identity and both proteins have C-terminal truncations (41). Because of these similarities, it is highly probable that ObgE*_Ca_* and ObgE*_Ct_* share functional characteristics. The ability of the *C. abortus* ObgE ortholog to interact with the *E. coli* 50S ribosomal subunit suggests that the function of ObgE in *Chlamydia* relates to ribosome biogenesis as documented in other bacteria (12, 42). As translation is essential for both replication and formation of infectious EBs, ectopic overexpression and knockdown of ObgE/*obgE* likely perturb the presence of functional ribosomes needed to support replication and EB formation. Dependence of ObgE on other proteins as well as GTP/GDP for normal function may explain why ectopic overexpression and knockdown do not produce opposing phenotypes.

**Fig 10:**
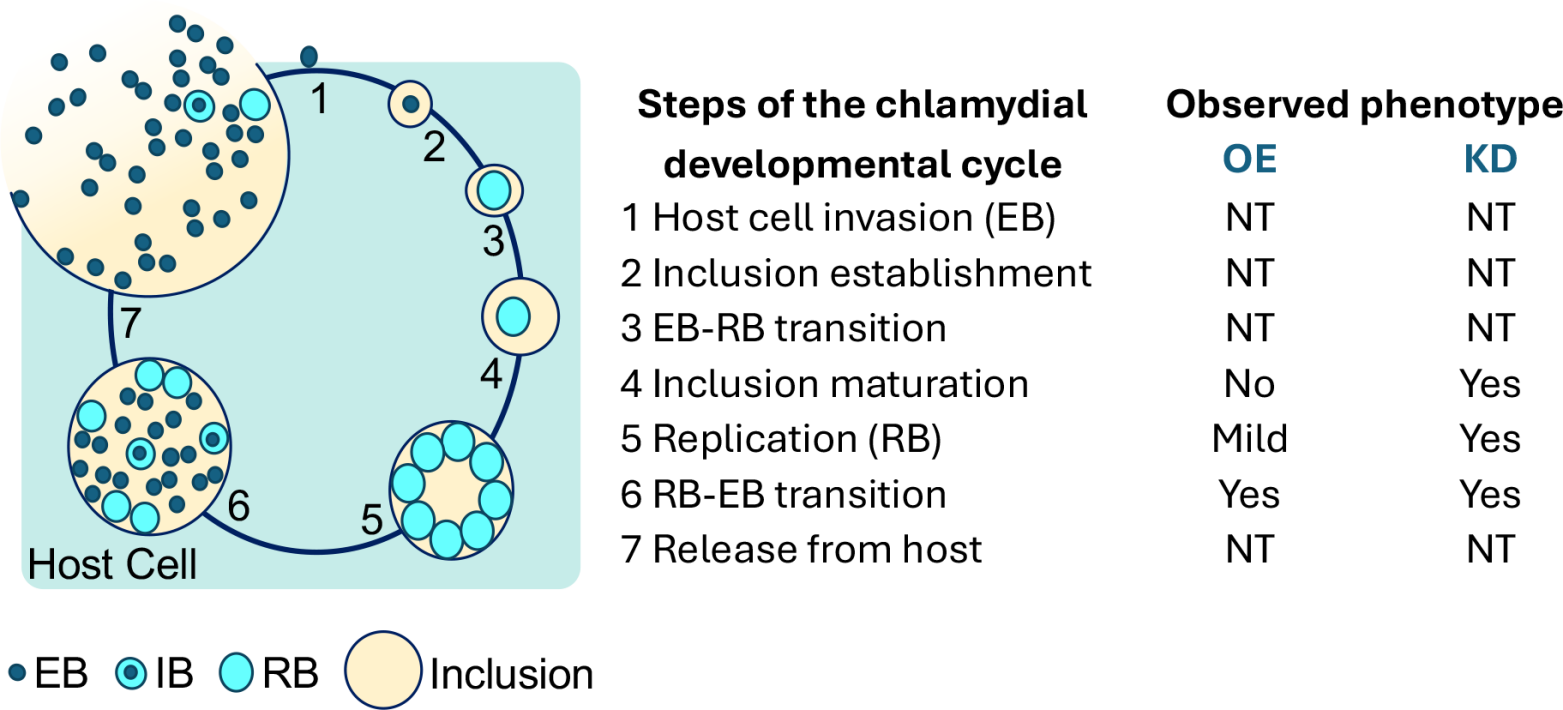
The effects of overexpression or knockdown of ObgE/*obgE* on seven steps during the *C. trachomatis* developmental cycle. A fine balance of ObgE expression is needed for optimal replication and development of *C. trachomatis*. Overexpression of ObgE resulted in decreases in recovered EBs, likely as the result of reduced transcription of EB-associated T3SS genes. On the other hand, insufficient expression of *obgE* is detrimental to RB replication, formation of infectious EBs, and inclusion development. OE = overexpression. KD = knockdown. NT = not tested.

Overall, this study shows that a balance in expression of ObgE is needed for proper progression through the *C. trachomatis* developmental cycle (Fig. 10). While the exact function of ObgE in *C. trachomatis* is unclear, we conclude that ObgE plays a vital role during the *C. trachomatis* developmental cycle.

## MATERIALS AND METHODS

### Organisms and cell culture

*Chlamydia trachomatis* serovar L2/434/Bu was propagated in the *Homo sapiens* cervical adenocarcinoma HeLa cell line (ATCC; Manassas, VA). Infected HeLa cells were cultured in RPMI 1640 with 2.05 mM glutamine (Hyclone; Logan, UT), additionally supplemented with 10% fetal bovine serum (FBS, HyClone; Logan, UT), 10 µg/mL gentamicin (MilliporeSigma; St. Louis, MO), and 1 µg/mL cycloheximide (MilliporeSigma; St. Louis, MO) at 37°C in a humidified CO_2_ (5%) incubator. EBs were purified by density gradient purification as described (43) following 48 h of infection and were stored at −80°C in sucrose phosphate glutamate (SPG) buffer (44).

### Vector construction

pBOMB4::R-ObgE-3xFLAG and pBOMB4::R-Clover-3xFLAG were made using the pBOMB4 backbone (45). Primers designed according to In-Fusion HD cloning (Takara Bio, USA) guidelines were used to linearize pBOMB4::R-DksA-3xFLAG (46) such that all elements of the vector were amplified (except the gene *dksA*). Genomic DNA was used as a template to generate the In-Fusion HD cloning insert specific to *obgE*. The In-Fusion HD cloning insert specific to Clover was amplified from p2TK2-SW2::E-Riboswitch-Clover (47). Clover, a variant of green fluorescent protein, was included as a control to ensure that phenotypic effects observed upon induction of R-ObgE are not the result of nonspecific effects of protein expression. The linearized vector and insert were ligated using the In-Fusion HD Cloning Kit (Takara Bio, USA) and transformed into stellar competent cells. p2TK2-SW2::dCas12-*obgE*_gRNA-PsciEng was constructed using the p2TK2-SW2 backbone (48). p2TK2-SW2::dCas12-*obgE*_gRNA-PsciEng was derived from the pBOMBL12CRia::L2 (incA_IGR) vector, a generous gift from Scot Ouellette (49). In short, the existing guide RNA targeting the gene *incA*, the aTC promoter, and the dCas12vaa coding sequence were inserted into the p2TK2-SW2_PsciEng vector (23) to create p2TK2-SW2::dCas12-*incA*_gRNA-PsciEng. The vector backbone and the necessary CRISPRi elements were amplified with PCR so that the *incA* guide RNA sequence was removed and replaced with a guide RNA targeting 20 bp in the 5′ promoter region of *obgE* to generate p2TK2-SW2::dCas12-*obgE*_gRNA-PsciEng. This vector was transformed into competent *E. coli* DH5α. Transformants were recovered from LB agar plates containing 100 µg/mL of carbenicillin or spectinomycin (Chem-Impex International; Wood Dale, IL). Plasmids were isolated using the PureLink Quick Plasmid Miniprep Kit (Invitrogen; Carlsbad, CA) and sequenced. Sequence-confirmed plasmids were isolated using the ZymoPure II Plasmid Maxiprep Kit (ZymoResearch; Irvine, CA). Plasmids were re-sequenced prior to transformation into *C. trachomatis*. All primers used in vector construction are described in Table S2.

### Transformation of *C. trachomatis*

*C. trachomatis* was transformed essentially as described (46), except for the use of water lysis to release transformed bacteria from host cells (45, 50). In short, crudely purified EBs were incubated with ~25 µg plasmid DNA and 600 µL of CaCl_2_ buffer (10 mM Tris pH [7.5], 50 mM CaCl_2_) for 30 min. After 30 min, antibiotic-free RPMI 1640 supplemented with 10% FBS was added to the transformation mix. Transformed bacteria were used to infect HeLa cell monolayers via centrifugation at 900* × g* for 15 min followed by incubation at 37°C, 5% CO_2_ for 48 h. At 18hpi, the medium was replaced with medium containing 0.3 µg/mL of penicillin G or 500 µg/mL of spectinomycin. Cultures with positive transformants were identified by fluorescence microscopy. Positive cultures were passaged for an additional two to three times to expand the population of transformants. Clonal populations were generated by five passages under limiting dilution. Plasmid DNA was isolated from clones and transformed into chemically competent *E. coli* TOP10. Four colonies were sequenced to confirm clonality.

### Analysis of ObgE expression

#### Immunofluorescence assay

HeLa cells were infected at an MOI of 5 with R-ObgE. Infected monolayers were fixed at 24hpi with 4% paraformaldehyde, permeabilized with 0.25% Triton-X, and blocked in 3% BSA. Monolayers were then incubated with M2 anti-FLAG antibody (MilliporeSigma; St. Louis, MO), washed with PBS, and then incubated with IgG rabbit anti-mouse-FITC (MilliporeSigma; St. Louis, MO). Monolayers were imaged for GFP using a Leica DMi8 inverted microscope (Leica Microsystems; Buffalo Grove, IL) equipped with an X-Cite LED light source and a DMC2900 Camera. Microscope settings that minimized background signal in samples with non-induced R-ObgE were used and kept consistent between experiments.

#### Western blot

Protein lysates were obtained from bacteria crudely purified from HeLa cells at 24–45hpi. Bacteria were lysed in Laemmli buffer containing β-mercaptoethanol (Bio-Rad; Hercules, CA) and heat denatured at 100°C for 10 min. 12% sodium dodecyl-sulfate polyacrylamide gel electrophoresis (SDS-PAGE) gels were loaded with lysate, normalized to 1 × 10^7^ GE per well. SDS-PAGE gels were electrophoresed at 40 mA and then transferred to a 0.45 µm PVDF membrane using a Trans-Blot Turbo transfer apparatus (Bio-Rad; Hercules, CA). The PVDF membrane was blocked in PBS with 0.1% Tween-20 and 3% nonfat dry milk. The membrane was probed with the M2 monoclonal anti-FLAG antibody (MilliporeSigma; St. Louis, MO), washed with PBS, and subsequently probed with goat anti-mouse HRP secondary antibody (Invitrogen; Carlsbad, CA). The membrane was treated with SuperSignal West Femto Luminol and Peroxide Solution (ThermoFisher; Waltham, MA) and imaged using a ChemiDoc Imaging System (Bio-Rad; Hercules, CA). Densitometry was done using Image-J (The National Institute of Health; Bethesda, MD).

### Analysis of GE and IFU in response to ectopic expression or knockdown of ObgE in *C. trachomatis*

HeLa cells were cultured to confluency in T-75 cm^2^ flasks, the cell culture medium was removed, replaced with HBSS, and inoculated with either WT, R-ObgE, R-Clover, or dCas12-*obgE* at an MOI of 5. Infected cultures were rocked for 15 min at RT and washed 3× with 100 µg/mL Heparin sodium salt dissolved in HBSS. The heparin wash was replaced with RPMI 1640 supplemented with 10% FBS (Hyclone; Logan, UT), 10 µg/mL gentamycin, 1 µg/mL cycloheximide, and expression was induced with 0.1 mM theophylline (ThermoFisher; Waltham, MA) or 0.5 nM aTC (MilliporeSigma; St. Louis, MO). Culture flasks were incubated at 5% CO_2_ for either 24 or 45hpi. At the time of harvest, cultures were washed 1× with 10 mL of K36 buffer (51) before infected monolayers were disrupted. Infected monolayers were scraped in 10 mL of K36 buffer and Dounce homogenized to gently release bacteria from the HeLa cells. To remove host debris, lysates were processed via two successive slow (500* × g*) and high (20,000* × g*) speed centrifugation steps at 4°C. Samples were resuspended in 35 µL of SPG buffer and stored at −80°C until GE analysis and IFU assays were performed. Data are presented as percent of the control. IFU assays were conducted using HeLa cells grown to confluency in 96-well plates with inocula normalized to GE.

### Microscopy

Micrographs for IFU assays were acquired using a Leica DMi8 inverted microscope (Leica Microsystems; Buffalo Grove, IL) equipped with an X-Cite LED light source and a DMC2900 Camera. *C. trachomatis* IFU assay plates were fixed with 100% methanol at 30hpi and stained with a fluorescein isothiocyanate-tagged anti-MOMP antibody (Thermo Fisher; Waltham, MA). Images were analyzed and processed with Image-J (The National Institute of Health; Bethesda, MD). Contrast and signal intensity were adjusted equally to the entire image. For TEM, infected HeLa cells were detached from T-175 cm^2^ cell culture flasks with 3 mL of Trypsin EDTA (MilliporeSigma; St. Louis, MO), pelleted at 1,500 *× g* for 3 min and fixed (2% glutaraldehyde, 2% paraformaldehyde, and 0.1M phosphate buffer) overnight at 4°C. Fixed samples were then post-fixed in 1% osmium tetroxide. Samples were dehydrated in a graded ethanol series and embedded in Spurr resin. Cross sections were obtained with an ultramicrotome (Riechert Ultracut R; Leica). Ultrathin sections were placed on formvar-coated copper grids and stained with 2% uranyl acetate and Reynold’s lead and observed on a Tecnai G2 transmission electron microscope (FEI Company; Hillsboro, OR).

### RNA extraction and analysis of gene expression

HeLa cells were cultured to confluency in T-75 cm^2^ flasks, the cell culture medium was removed, replaced with HBSS and inoculated with either WT, R-ObgE, R-Clover, or dCas12-*obgE* at an MOI of 5. To determine if ectopic overexpression of ObgE affected the expression of selected genes, 0.1 mM of theophylline was added to infected cell culture flasks at the time of infection, and RNA was isolated as previously described (46) at 24 and 45hpi. To determine if *obgE* knockdown affected the expression of selected genes in a timepoint-dependent manner, a timecourse experiment was performed in which knockdown of *obgE* was induced with 0.5 nM of aTC at 5, 15, 25, and 35hpi and RNA was isolated as previously described (46) 10 h after induction (15, 25, 35, and 45hpi). In short, infected HeLa cells were detached by scraping in 1 mL of ice-cold K36 buffer. Samples were pelleted at 20,000* × g* for 5 min at 4°C. Pellets were resuspended in 100 µL of ice-cold SPG buffer. A small portion of each sample was removed for GE analysis. The remainder of the sample was mixed with 1 mL TRIzol Reagent (Invitrogen, USA) and mechanically disrupted with 0.1 mm zirconia beads at 4 m/s for 20 s, twice with a FastPrep 24 Tissue Homogenizer (MP Biomedicals; Santa Ana, CA). Total RNA was extracted from TRIzol using chloroform, isopropanol, and ethanol precipitation according to manufacturer guidelines. Isolated RNA was treated with DNase I (Thermofisher; Waltham, MA) and cleaned with the Zymo RNA Clean and Concentrate-5 Kit. RNA integrity was verified by an Agilent 2100 Bioanalyzer and the RNA 6000 Pico Kit (Agilent Technologies; Santa Clara, CA). RNA (2,000 µg) was converted into cDNA using the SuperScript III First Strand Synthesis Kit (Invitrogen; USA). cDNA was diluted 10-fold and 20 ng of cDNA (2 µL) was used for each primer pair reaction. Transcripts/mL of each gene of interest were determined via qPCR by fitting the Cq values to standard curves prepared from genomic DNA for each primer pair. Transcripts were normalized to GE to account for differences in the number of bacteria from which RNA was extracted. GE were quantified via qPCR using a standard curve generated from a plasmid containing a single copy of the chlamydial gene *hctA*. Fold change values were quantified by dividing the induced normalized transcript/mL by the non-induced normalized transcript/mL for each sample type and timepoint. Primers used for transcriptional analysis are described in Table S3.

### Statistical analysis

Statistical analyses were conducted using Prism (GraphPad Inc.; San Diego, CA) software.

## SUPPLEMENTAL MATERIAL

Supplemental materials jb.00282-25-s0001.pdf Supplemental methods, Figures S1 to S4, and Tables S1 to S3.

ASM does not own the copyrights to Supplemental Material that may be linked to, or accessed through, an article. The authors have granted ASM a non-exclusive, world-wide license to publish the Supplemental Material files. Please contact the corresponding author directly for reuse.
