# Supplementary material for: Ectopic overexpression and CRISPRi-based knockdown of Chlamydia trachomatis ObgE inhibits RB replication and EB reformation
Source: J Bacteriol. 2025 Nov 12;207(12):e00282-25. doi: 10.1128/jb.00282-25 (PMC12713372; doi:10.1128/jb.00282-25)
Supplement: Supplemental materials — Supplemental methods, Figures S1 to S4, and Tables S1 to S3. [file jb.00282-25-s0001.pdf]

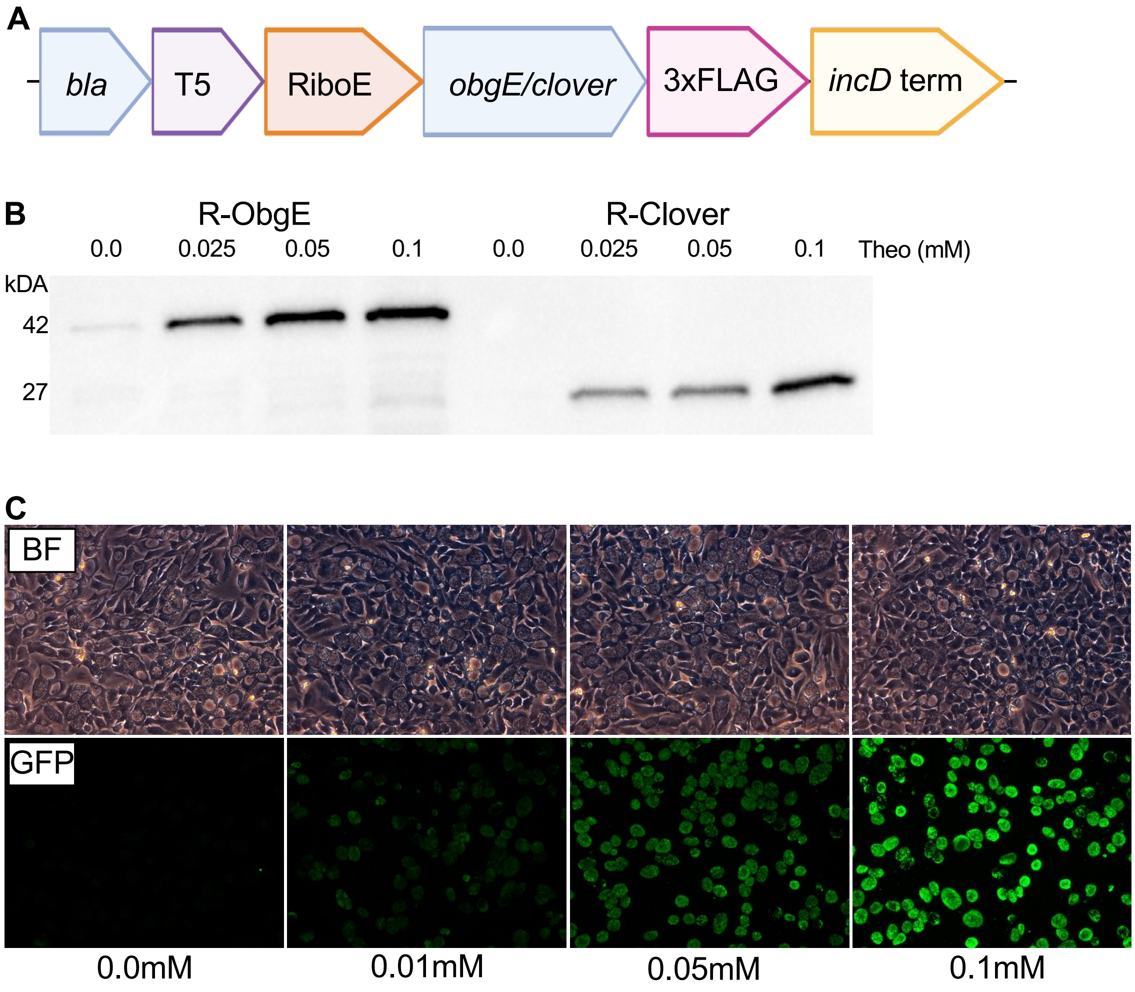

Supplemental Figure 1. Confirmation of ectopic overexpression of ObgE-3xFLAG. (**A**) Schematic of R-ObgE and R-Clover consisting of a beta-lactam antibiotic resistance gene (*bla*), T5-lac promotor (T5), E riboswitch (RiboE), the *C. trachomatis obgE* or *clover* genes, and an in-frame 3xFLAG-tag. (**B**) Expression of ObgE-3xFLAG and Clover-3xFLAG at 24hpi is dependent on the concentration of Theophylline (Theo). All samples were normalized to $1x10^7$ GE. The expected sizes of ObgE-3xFLAG and Clover-3xFLAG are 42kDA or 27kDA, respectively. (**C**) Immunofluorescence assay shows that expression ObgE-3xFLAG at 24hpi depends on the concentration of Theo.

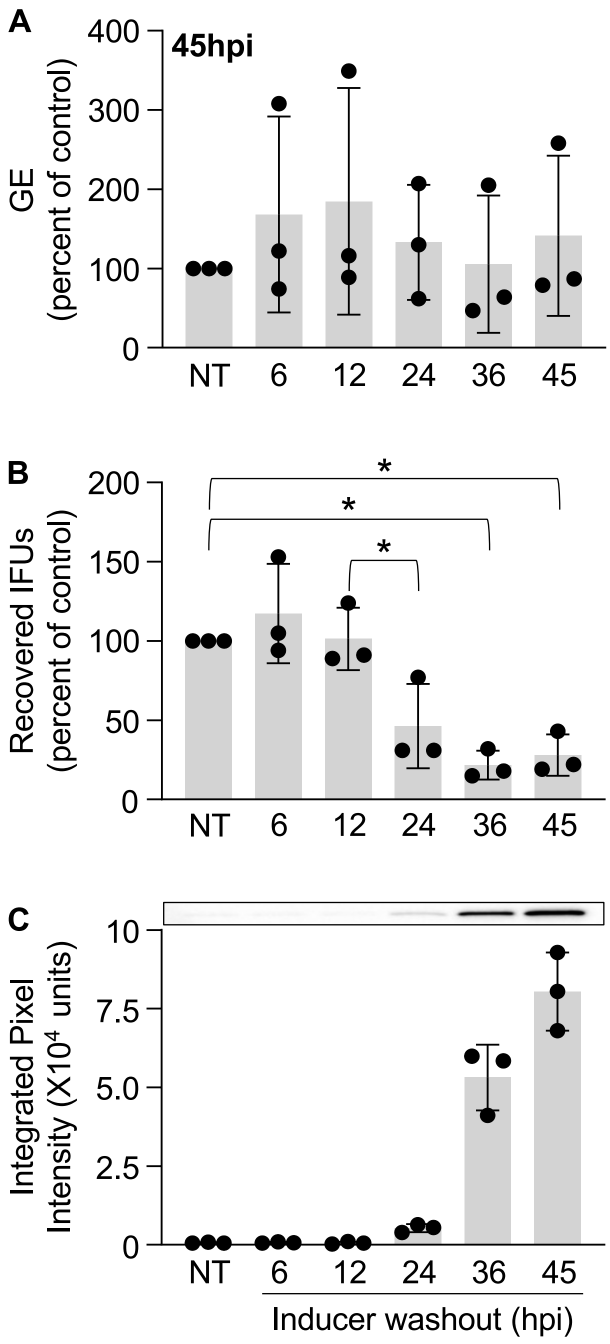

Supplemental Figure 2. Removal of Theophylline from the culture medium shows that the effects of ObgE ectopic overexpression are restricted to late time points. (**A**) Removal of Theophylline at different time points during incubation had no effect on GE. (**B**) Recovery of IFUs was only affected when Theophylline was removed from the culture medium at 36hpi or if not removed at all. (**C**) Western blot analysis for expression of ObgE-3xFLAG at 45hpi. The blot insert is representative of three independent experiments. Integrated pixel intensity was calculated by Image-J. Samples for IFU and SDS-PAGE analysis were normalized to GE. NT = No treatment. Bars=Mean ±SD (N=3). Statistical significance was calculated using One-Way ANOVA with Tukey's post-hoc test. P<0.05 was considered significant.

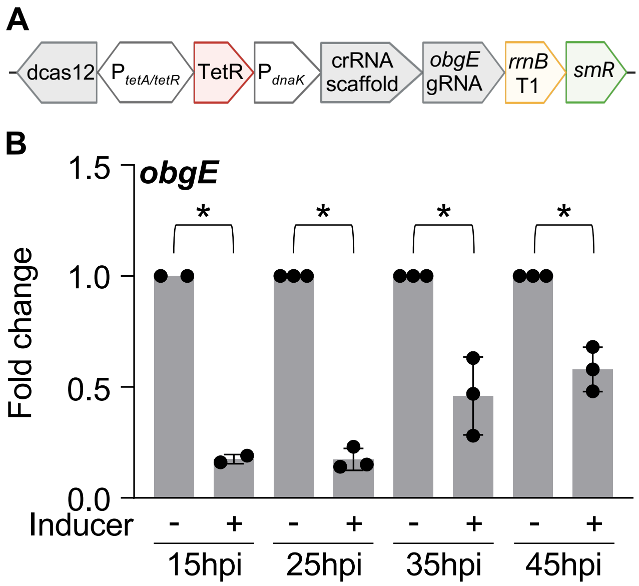

Supplemental Figure 3. Confirmation of knockdown of *obgE.* (**A**) Schematic of dCas12-*obgE* consisting of the spectinomycin antibiotic resistance gene (*smR*), CRISPRi machinery, and a guide RNA targeting the 5' UTR region of *obgE.* (**B**) Transcriptional analysis of *obgE* at 15, 25, 35, and 45hpi. *obgE* transcripts are significantly reduced at each timepoint. Bars=Mean $\pm$ SD (N≥2). Statistical significance was calculated using One-Way ANOVA with Tukey's post-hoc test. $P<0.05$ was considered significant.

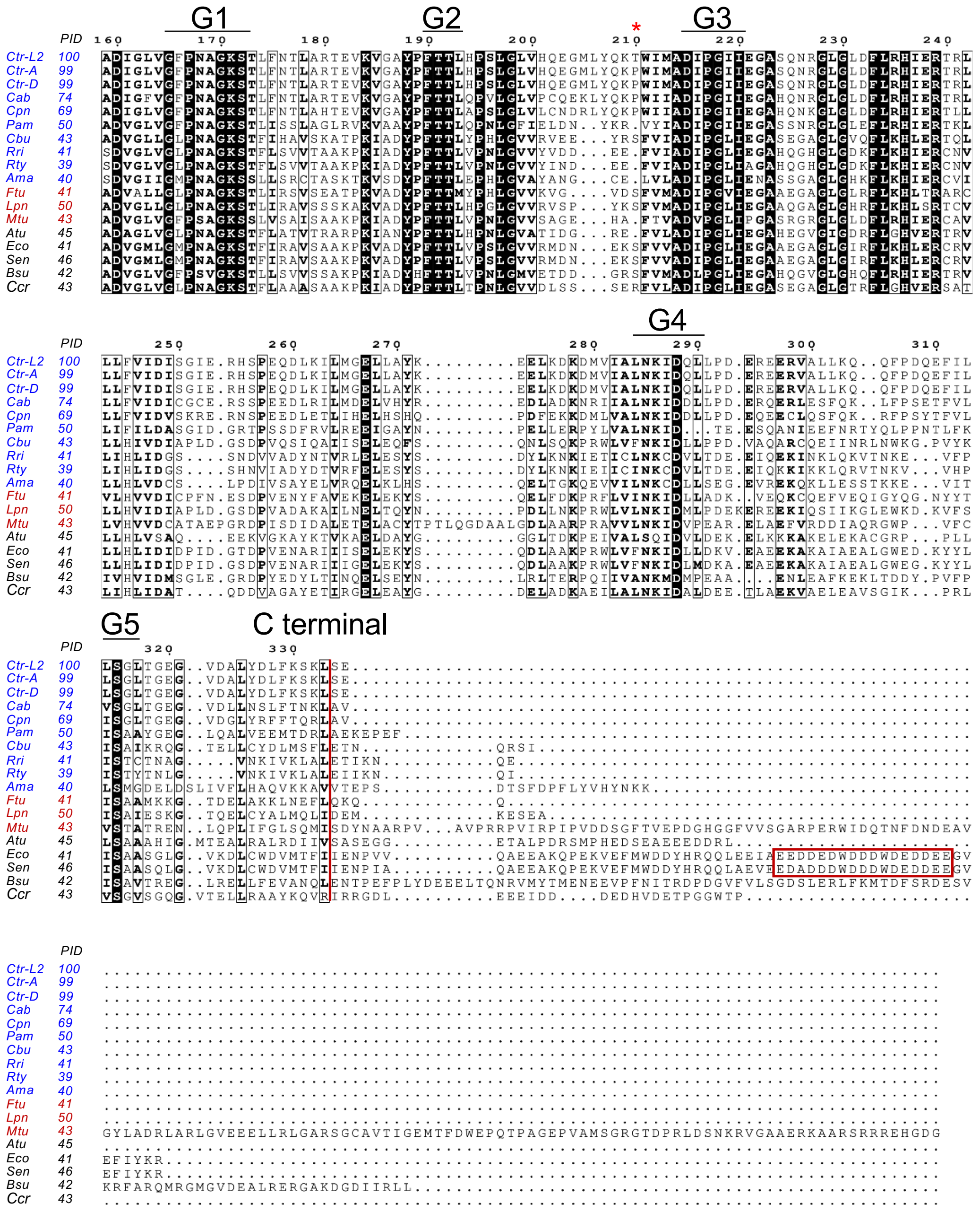

Supplemental Figure 4. Multiple sequence alignment of G-domain and C-terminal regions of ObgE orthologs. *Chlamydia trachomatis* (*Ctr*) serovars L2, A, and D, *Chlamydia abortus* (*Cab*), *Chlamydia pneumoniae* (*Cpn*), *Protochlamydia amoebophila* (*Pam*), *Coxiella burnetii* (*Cbu*), *Rickettsia rickettsii* (*Rri*), *Rickettsia typhi (Rty), Anaplasma marginale* (*Ama*)*, Legionella pneumophila* (*Lpn*), *Francisella tularensis* (*Ftu*), *Mycobacterium tuberculosis* (*Mtu*), *Agrobacterium tumefaciens* (Atu), *Escherichia coli* (*Eco*), *Salmonella enterica* (*Sen*), *Bacillus subtilis* (*Bsu*), *Caulobacter crescentus* (*Ccr*). White characters on a black background represent conserved residues. Bolded black characters on a white background represent similar residues. Red line indicates the start of the C-terminal region. The asterisk indicates a sequence difference among the *Chlamydia* ObgE homologs. The red rectangle indicates residues that mediate interaction with YbiB in *E. coli* with similar residues in *S. enterica* included for comparison. Obligate intracellular bacteria (Blue); facultative intracellular bacteria (Red); extracellular bacteria (Black). PID = percent identity.

Supplemental Table 1: **Annotation of *ybiB*/YbiB in select bacteria**

**Bacterium**	**taxid**	**Similar gene or protein detected**	**Percent identity to *E.coli ybiB***
Chlamydia trachomatis	813	No	N/A
Chlamydia abortus	83555	No	N/A
Protochlamydia amoebophila	362787	No	N/A
Chlamydia pneumoniae	83558	No	N/A
Coxiella burnetti	777	No	N/A
Rickettsia rickettsii	783	No	N/A
Rickettsia typhi	785	No	N/A
Anaplasma marginale	770	No	N/A
Francisella tularensis	263	No	N/A
Legionella pneumophila	446	No	N/A
Mycobacterium tuberculosis	1773	No	N/A
Agrobacterium tumefaciens	358	No	N/A
Bacillus subtilis	1423	No	N/A
Caulobacter crescentus	155892	No	N/A
Salmonella enterica	28901	Yes	78%

Supplemental Table 2: **Primers used for vector construction**

**Primer Name**	**Sequence 5’→3’**	**Purpose**
Ribo-pBOMB4_For	ATGGACTACAAAGACCATGACGGT	Linearization of R-DksA-3xFLAG vector
Ribo-pBOMB4_Rev	CTTGTTGTTACCTCCTTAGCAGGG	linearization of R-DksA-3xFLAG vector
obgE_For	GGAGGTAACAACAAGATGTTTGTT<br>GATCAAATTACATTAGAGTTGCGC	Amplify *obgE* for InFusion-HD cloning ligation
obgE_Rev	GTCTTTGTAGTCCATCTCACTGAG<br>TTTACTCTTGAACAGATCATACAA	Amplify *obgE* for InFusion-HD cloning ligation
Clover_For	GGAGGTAACAACAAGATGGTGAG<br>CAAGGGCGA	Amplify *clover* for InFusion-HD cloning ligation
Clover_Rev	GTCTTTGTAGTCCATGTCGACCTT<br>GTAGAGCTCGTCC	Amplify *clover* for InFusion-HD cloning ligation
Ribo-confirm_For	TACCAGCATCGTCTTGATGCCCTT<br>GG	Confirmation of successful InFusion-HD ligation of *obgE* fragment and linearized vector
obgE-confirm_Rev	TCCAATCCCAATCCCCGGTTTTGCG	Confirmation of successful InFusion-HD ligation of *obgE* fragment and linearized vector
obgE-seq_For	AATTGATACACGATTTCACC	Confirmation of *obgE* sequence
obgE-seq_Rev	TGACCATGTCTTTATCCTTT	Confirmation of *obgE* sequence
obgE_gRNA	AGCTTGAACAAACTGAATTA	20bp guide RNA sequence
obgE_gRNA_For	5’PO_4-AACTGAATTAATCTACAA<br>GAGTAGAAATTAGGTGTC	Inverse PCR to replace the *incA*_gRNA with *obgE*_gRNA. Sequence specific to the guide RNA is underlined

obgE_gRNA_Rev	5’PO_4-TGTTCAAGCTCAAATAAAACGAAAGGCTCAGTCG	Inverse PCR to replace the *incA*_gRNA with *obgE*_gRNA. Sequence specific to the guide RNA is underlined
PsciEng-Vector_For	AGCTGCTTAAGACGGATCCCTTGTACAATCAATTTACCGATTAAATAGTCTC	Linearize p2TK2-SW2_PsciEng vector
PsciEng-Vector_Rev	GAGCTAGTGCCTATTTATACAGTTCATCCATGCCCATCACATCGGTAAACG	Linearize p2TK2-SW2_PsciEng vector
5'Cas12 system_For	GTATAAATAGGCACTAGCTCAGATTCAGTAGACCGCTGTTGATAAAACG	Amplify dCas12 system
3'Cas12 system_Rev	GGGATCCGTCTTAAGCAGCTACGTTGCGCAGCTCC	Amplify dCas12 system

Supplemental Table 3: **Primers used for analysis of GE and gene expression**

**Application**	**Gene**	**Forward Primer 5'→3'**	**Reverse Primer 5'→3'**
GE analysis	*hctA*	AGCAAAGCCGCTGCTAAA	TTTGTTGGTTTGACCTTTGC
Developmental kinetics	*euo*	CCCAACGAGTAGTCTTCGATGC	CTCAGGCTGCCAAGTTGCA
Developmental kinetics	*porB*	TTCATTAGACGCTATGCCTGCG	TTCAGGTTACCGGACAGTGCG
Developmental kinetics	*hctB*	CCGTAGCAGTAAAAGCTGGAGTTT	GAGCTGTACGAGAACGGTTAGG
Knockdown confirmation	*obgE*	CAGCGTTAGGGAATCCAACGAG	CCGAGCTCCAACAAAAGCTACT
T3SS	*tarp*	CTTCGGCATCAGGTGCTTCC	GCCGCTGAATTTGTTGCGTT
T3SS	*scc2*	GGCCGAGATTGCTGCACAAA	CCCCTGTAGGATGTCCATGAGA
T3SS	*CLT0238*	CTTTCCTCCGTACCGACAAACG	GAGACAGAACAACGTCTCCTATGC
T3SS	*incA*	TCTGATCGCTCCACAAATCAC	CTTCTCTTTGCAGATCCTGGTATA
T3SS	*incD*	GTTGTTGCGGGCTTGTTGTT	ACTCCTCCAACCAAAGCTCC
T3SS	*cpoS*	ATCGGGACCCCAGCTTCT	GACAACTTCTAACTGACGCTCAATC
Polar effect	*rpmA*	TGCAGGATGCCATTTGGTTCCT	GTCAGGGAGCAAGCCGTAAC
Polar effect	*rplU*	ACCAGGTTCGCAAAGGTGAC	TCTCCTTTCACTACAGCGTTGC
Polar effect	*CTL0674*	AGCCTGTCGTTTCTCCTACAAAATG	TGACAGGCCCAACTGGCAA

Ectopic overexpression and CRISPRi-based knockdown of *Chlamydia trachomatis* ObgE inhibits RB replication and EB reformation

Colleen C. Monahan[1], Kiara Held[1], Hong Yang[1], Geselle Sotelo[1], Nicole Grieshaber[2], Scott Grieshaber[2], and Anders Omsland[1*]

SUPPLEMENTAL MATERIALS AND METHODS

Amino acid alignments and BLAST search

Multiple sequence analysis of ObgE G-domains and C-terminal domains was performed using the ClustalW algorithm (1) in MEGA Software (2). The multiple sequence alignment was viewed with ESPript3.0 (3). ObgE amino acid sequences included in this analysis: *Chlamydia trachomatis* serovar L2/434/Bu (B0B7Y8), *C. trachomatis* serovar A (Q3KLT5), *C. trachomatis* serovar D (O84423), *C. abortus* (Q5L6R9), *C. pneumoniae* (A0A0F7WZ05), *Protochlamydia amoebophila* (A0A0U5EPN8), *Coxiella burnetii* (Q83ED8), *Rickettsia rickettsii* (B0BVJ1), *Rickettsia typhi* (*Q68VS1), Anaplasma marginale* (B9KIJ7)*, Legionella pneumophila* (A0AAN2RPY5), *Francisella tularensis* (A4IVW3), *Mycobacterium tuberculosis* (A0A0H3LFX0), *Escherichia coli* (A0A8A5IF75), *Salmonella Enterica* (A0A3V4QYI8), *Agrobacterium tumefaciens* (A0A822UYJ5), and *Caulobacter crescentus* (B8GYI7). For the BLAST search with *E. coli ybiB* (NC_000913.3), query subrange:835248-836210 was used as the query sequence. *ybiB* was blasted against the genomes listed in Supplemental Table 1. A cut off value of E^{-7} was used.

REFERENCES

1. Thompson JD, Higgins DG, Gibson TJ. 1994. CLUSTAL W: improving the sensitivity of progressive multiple sequence alignment through sequence weighting, position-specific gap penalties and weight matrix choice. Nucleic Acids Res 22:4673-80.

2. Kumar S, Stecher G, Suleski M, Sanderford M, Sharma S, Tamura K. 2024. MEGA12: Molecular Evolutionary Genetic Analysis version 12 for adaptive and green computing. Mol Biol Evol 41.

3. Robert X, Gouet P. 2014. Deciphering key features in protein structures with the new ENDscript server. Nucleic Acids Res 42:W320-W324.
